# Laparoscopic cholecystectomy under spinal anaesthesia *vs*. general anaesthesia: a meta-analysis of randomized controlled trials

**DOI:** 10.1186/s12871-015-0158-x

**Published:** 2015-12-03

**Authors:** Gan Yu, Qin Wen, Li Qiu, Li Bo, Jiang Yu

**Affiliations:** 1 Department of Hepatobiliary Surgery, The Affiliated Hospital of Luzhou Medical College, 25 Taiping Road, Luzhou City, Sichuan Province 646000 P.R. China; 2 Department of infectious diseases, The Affiliated Hospital of Luzhou Medical College, 25 Taiping Road, Luzhou City, Sichuan Province 646000 P.R. China

**Keywords:** Cholecystectomy, Laparoscopic, Cholecystectomy, Laparoscopy, Regional anesthesia, Spinal anesthesia

## Abstract

**Background:**

Laparoscopic Cholecystectomy (LC) is conventionally performed under general anaesthesia (GA), but there are multiple studies which have found spinal anaesthesia (SA) as a safe alternative. This meta-analysis was performed after adding many recent randomized controlled trials (RCTs) to clarify this issue.

**Methods:**

Relevant articles published in English were identified by searching PubMed, Embase, Web of Knowledge, and the Cochrane Controlled Trial Register from January 1, 2000 to December 1, 2014. Reference lists of the retrieved articles were reviewed to identify additional articles. Primary outcomes (postoperative pain scores) and secondary outcomes (operating time (OT) and postoperative complications) were pooled. Quantitative variables were calculated using the weighted mean difference (WMD), and qualitative variables were pooled using odds ratios (OR).

**Results:**

Seven appropriate RCTs were identified from 912 published articles. Seven hundred and twelve patients were treated, 352 in SA group and 360 in GA group. LC under SA was superior to LC under GA in postoperative pain within 12 h (visual analogue score (VAS) in 2–4 h, WMD = −1.61, *P* = 0.000; VAS in 6–8 h, WMD = −1.277, *P* = 0.015) and postoperative complications (postoperative nausea and vomiting (PONV) WMD = 0.427, *P* = 0.001; Overall Morbidity WMD = 0.691, *P* = 0.027). The GA group was superior to SA group in postoperative urinary retention (WMD = 4.273, *P* = 0.022). There were no significant differences in operating time (WMD = 0.184, *P* = 0.141) between two groups.

**Conclusions:**

SA as the sole anaesthesia technique is feasible, safe for elective LC.

## Background

Laparoscopic cholecystectomy (LC) has become the gold standard for the surgical treatment of symptomatic cholelithiasis and has gained worldwide acceptance [1]. It is a minimally invasive procedure with a significantly shorter hospital stay and a quicker convalescence compared with the classical open cholecystectomy [2].

LC is conventionally done under general anaesthesia (GA) and may be associated with postoperative pain and nausea and vomiting (PONV). Rodgers et al., published a meta-analysis showing that the use of neuraxial techniques for a variety of surgical procedures resulted in a decrease in mortality, venous thromboembolism, myocardial infarction, and several other complications [3]. Spinal anesthesia (SA) is a commonly used anaesthetic technique that has a very good safety profile. SA has several advantages over GA. These advantages include the patients’ being awake and oriented at the end of the procedure, less postoperative pain, and the ability to ambulate earlier than patients receiving general anesthesia. Moreover, the incidences of nausea and vomiting are less with selective spinal anesthesia than with general anesthesia [4]. SA is more effective than GA in blunting the neuroendocrine stress and adverse responses to surgery [5]. Some possible problems related to the technique of general anesthesia such as teeth and oral cavity damage during laryngoscopy, sore throat, and pain related to intubation and/or extubation are prevented by administering selective spinal anesthesia to patients undergoing laparoscopic interventions [6]. There are multiple reports that have been published regarding the feasibility of SA for LC in patients fit for GA [7–13].

Surprisingly, in the era of minimally invasive medicine, regional anesthesia has not gained popularity and has not been routinely used as a sole method of anesthesia in laparoscopic procedures. Johnson noted that “all laparoscopic procedures are merely a change in access and still require general anesthetic; hence the difference from conventional surgery is likely to be small” [14]. This statement is predominantly based on the assumption that laparoscopy necessitates endotracheal intubation to prevent aspiration and respiratory distress secondary to the induction of carbon dioxide pneumoperitoneum, which is not well tolerated in a patient who is awake during the procedure [4, 15, 16].

These contradictions make it necessary to more closely compare SA and GA in LC, to evaluate whether SA in LC is associated with better results. This comprehensive meta-analysis included many recent randomized controlled trials (RCTs) and was systematically conducted to verify whether SA superior to GA for laparoscopic cholecystectomy or not.

## Methods

A meta-analysis protocol was drafted before the initial search was started. The meta-analysis was conducted and reported according to the Preferred Reporting Items for Systematic Reviews and Meta-Analyses (PRISMA) statement issued in 2009 [17].

### Literature search

We searched PubMed, Web of Knowledge, Embase and the Cochrane Controlled Trial Register to identify relevant articles published from January 1, 2000 to December 1, 2014 using the search phrases (((((regional anaesthesia) OR regional anesthesia) OR spinal) OR general anesthesia)) AND (((((laparoscop* or celioscop* or coelioscop* or abdominoscop* or peritoneoscop*) AND cholecystecto* or colecystecto*)) OR “Cholecystectomy, Laparoscopic)). Appropriate adjustments were required according to the database. Filters were used in PubMed, Embase and Web of Knowledge to exclude animal and non-English studies. A manual search of published meta-analyses and relevant articles was performed to identify additional articles.

### Article selection

The process of article selection was based on the PRISMA flow diagram [15]. Selected studies met the following criteria: (a) RCT design; (b) compared SA and GA; (c) revealed at least one of the primary or secondary outcomes mentioned below; and (d) were published in English. Articles were excluded if: (a) the surgery was not cholecystectomy; (b) spinal anesthesia was not mentioned; (c) it was a retrospective study, prospective nonrandomized study, animal study, review, letter, meeting, or comment. When multiple published articles from the same study were available, the report with the most detailed information was selected.

### Data extraction

Primary outcomes evaluated included postoperative pain score. Pain scores from RCTs using a visual analogue scale/score (VAS) were pooled to assess postoperative abdominal pain. Three postoperative time points were used to evaluate pain, 2 to 4 h, 6 to 8 h, and 24 h.

Secondary measures evaluated included intraoperative outcomes (Operating time, Intraoperative Events), postoperative complications (Nausea and Vomiting, Urinary retention and Overall Morbidity). Intraoperative Events (events occurring during spinal anaesthesia included hypotension, right shoulder pain, Nausea and Vomiting).

Overall Morbidity is that the morbidity which each included studies reported were pooled by meta analyze.

Patient characteristics (number of patients, gender, age and body mass index) were also recorded. If the above data was not available in the published study, the authors were contacted and asked to supply the information.

### Assessment of study quality

The literature search, article selection, data extraction and assessment of study quality were completed independently by two authors (Gan and Qin). Discrepancies were resolved by discussion. When a consensus could not be reached, a third author (Li) broke the tie. Quality assessment was independently conducted in all the included studies by three investigators (Gan, Qi and Li.) using the Jadad’s revised rating scale [18]. Disagreements were resolved by discussion. Jadad’s revised rating scale comprised of four parameters of quality: Generate (0–2 points), Hide (0–2 points), Double Blinding (0–2 points) and Withdraws And Dropouts (0–1 points). The maximum possible score is 7 points and Jadad’s scores ≥4 are considered as high-quality studies.

### Statistical analysis

Continuous variables were combined using the weighted mean difference (WMD). The method of Hozo et al. [19] was used if variables were provided as medians or/and ranges instead of a mean with a standard deviation. Binary variables were pooled using an odds ratio (OR). Homogeneous data was evaluated using fixed effect models. The inverted variance method was used for continuous variables and the Mantel-Haenszel method for binary variables. Random effect models based on the DerSimonian & Laird method were used to calculate the combined outcomes of both continuous and binary variables when heterogeneity existed. *P* < 0.05 was considered statistically significant. Heterogeneity was identified using a chi-square-based Q-test (*P* ≤ 0.10) and *I*^2^ index (*I*^2^ exceeding 50 %). If heterogeneity was found, a meta-regression based on the Restricted Maximum Likelihood (REML) method was conducted to identify any related factors (*P* < 0.05 was considered significant). Subgroup analyses were conducted to identify potential sources of heterogeneity when the meta-regression was not adequate (less than 10 studies reported the outcome) or as a supplementary method. Sensitivity analyses were performed to examine the effect of excluding lower quality studies. Publication bias was evaluated using Egger’s regression test, with *P* < 0.05 indicating statistically significant publication bias. The confidence interval (CI) was established at 95 %. Statistical analyses were carried out using Stata12.0 software (Stata Corporation, USA).

## Results

### Identification of studies and quality of the RCTs

**Fig. 1:**
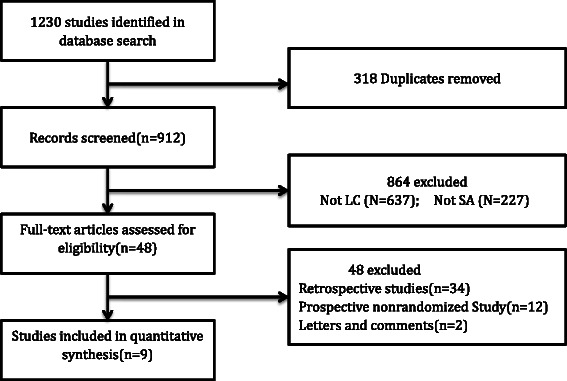
PRISMA flow diagram of the systematic article selection process

Nine RCTs [1, 10, 12, 20–25] were extracted from 1230 publications identified from databases and other sources. The PRISMA [15] flow diagram for this meta-analysis is presented in Fig. 1. Two studies [24, 25] were called randomized without defining the method of randomization or blinding and whether patients withdrew or dropped out. Only seven high-quality [1, 10, 12, 20–23] articles were included in the meta-analysis.

### Characteristics of included RCTS

Seven hundred and twelve patients (352 with SA, 360 with GA) were identified to be included in the meta-analysis. Table 1 shows the general characteristics, including sample size, M/F ratio, age, and body mass index (BMI). There was no significant difference in number of patients, Male/Female ratio, Age and BMI between groups.

**Table 1:** General characteristics of the 7 studies included in the meta-analysis

	Num		M/F ratio		Age (year)		BMI (kg/m^2^)	
Study	SA	GA	SA	GA	SA	GA	SA	GA
Kalaivani V. [20], 2014	23	25	10/15	8/17	45 ± 11.73^b^	47.84 ± 10.49^b^	–	–
Bessa SS. [1], 2012	86	90	11/79	8/82	40	44	28.7	29.1
					(16–50)^a^	(19–50)^a^	(22.8–34)^a^	(23.4–33.1)^a^
Imbelloni LE, [21], 2010	34	33	26/9	23/10	41.1 ± 12.4^b^	45.2 ± 12.1^b^	–	–
*Tzovaras* G. [10], 2008	49	48	29/20	30/18	44	46	25	26
					(23–65)^a^	(26–65)^a^	(18–30)^a^	(19–30)^a^
Ellakany M. [22], 2013	20	20	8/12	7/13	45.9 ± 13.6^b^	44.3 ± 13.2^b^	29.8 ± 4.1^b^	30.0 ± 3.9^b^
Bessa SS [23], 2010	30	30	5/25	6/24	41.4 ± 11.1^b^	40.9 ± 11^b^	31.3 ± 4.1^b^	30.8 ± 6.6^b^
Tiwari S [12], 2013	110	114	13/96	16/98	45.07 ± 13.19^b^	46.10 ± 12.9^b^	-	-

Data are expressed as mean ± standard deviation or as numbers

*BMI* body mass index

^a^Median (range); ^b^mean ± standard deviation

### Quantitative synthesis

#### Primary outcomes

**Fig. 2:**
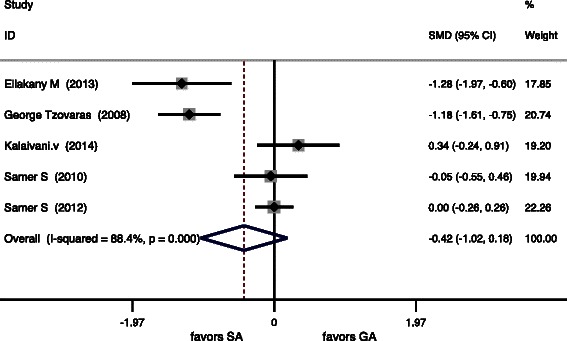
Forest plots for primary outcomes included postoperative pain scores in 24 h

**Fig. 3:**
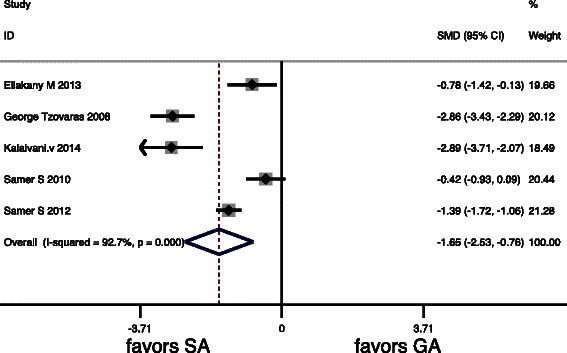
Forest plots for primary outcomes included postoperative pain scores in 2 h to 4 h

**Fig. 4:**
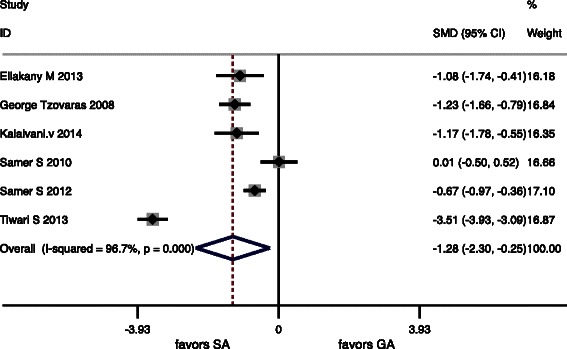
Forest plots for primary outcomes included postoperative pain scores in 6 to 8 h

The postoperative pain scores were assessed in 7 studies (Table 2). Table 3 summarizes the pooled results of the VASs. VASs from postoperative 2 to 4 h and 6 to 8 h were significantly lower after LC under SA (*P* = 0.000 and *P* = 0.015, respectively). There were no significant differences in VASs at 24 h (*P* = 0.17). Forest plots of primary outcomes are listed in (Figs. 2, 3 and 4).

**Table 2:** VASs of the 7 studies included in the meta-analysis

	VAS (2–4 h)		VAS (6–8 h)		VAS (24 h)	
Study	SA	GA	SA	GA	SA	GA
Kalaivani V. [20], 2014	0.45 ± 1.35^b^	4.16 ± 1.22^b^	3.55 ± 0.90^b^	4.92 ± 1.38^b^	3.80 ± 0.97^b^	3.48 ± 0.94^b^
Bessa SS. [1], 2012	4 (0–10)^a^	6 (3–10)^a^	4 (1–10)^a^	4 (1–10)^a^	-	-
Imbelloni LE [21], 2010	-	-	-	-	-	-
*Tzovaras* G. [10], 2008	0 (0–4)^a^	3 (0–8)^a^	0 (0–6)^a^	2 (0–7)^a^	0 (0–4)^a^	1 (0–6)^a^
Ellakany M. [22], 2013	1.2 ± 1.2^b^	2.3 ± 1.6^b^	1.6 ± 1.4^b^	3.4 ± 1.9^b^	0.8 ± 0.7^b^	2.3 ± 1.5^b^
Bessa SS [23], 2010	5 (0–8)^a^	5 (2–9)^a^	2 (1–6)^a^	3 (0–5)^a^	2 (0–4)^a^	2 (1–8)^a^
Tiwari S [12], 2013	-	-	1 (0–4)^a^	4 (1–7)^a^	0 (0–2)^a^	1 (0–4)^a^

Data are expressed as mean ± standard deviation/mean

*VAS* visual analogue scale/score

^a^Median (range). ^b^mean ± standard deviation

**Table 3:** Meta-analysis of the primary outcomes in 7 RCTs

Quantitative synthesis					Heterogeneity		Bias
Outcomes	OR (95 % CI)		z	*p*	I^2^	*P*	*P*
VAS (2–4 h)	−1.64	−2.54	3.62	*P* = 0.000	93 %	*P* = 0.000	0.61
		−0.76					
VAS (6-8 h)	−1,277	−2.3	2.44	*P* = 0.015	97 %	*P* = 0.000	0.94
		−0.25					
VAS (24 h)	−0.42	−1.07	1.37	*P* = 0.17	88 %	*P* = 0.000	0.51
		0.18

#### Secondary outcomes

**Fig. 5:**
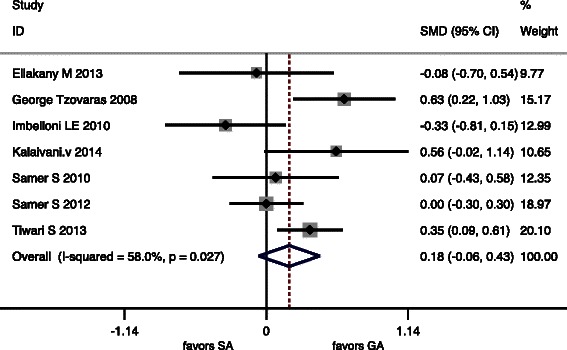
Forest plots for operating time

Seven studies reported OT (Table 4). There were no significant differences in OT (*P* = 0.14). Pooled results of OT are listed in Table 5. Seven studies reported Intraoperative Events (Table 4). In the SA group 6 studies reported shoulder pain. Five studies reported Nausea and Vomiting, Six studies reported Hypotension. Forest graphs for intraoperative outcomes are shown in Fig. 5.

**Table 4:** Intraoperative outcomes of the 7 studies included in the meta-analysis

	Operating time (min)		Intraoperative events in spinal anesthesia group		
Study	SA	GA	Shoulder pain	Nausea and vomiting	Hypotension
Kalaivani V1. [20], 2014	97.2 ± 34.08^b^	81.95 ± 20.97^b^	6	1	9
Bessa SS. [1], 2012	35 (20–78)^a^	35 (19–75)^a^	32	-	29
Imbelloni LE [21], 2010	62.9 ± 11.3^b^	66.8 ± 12.5^b^	16	1	14
*Tzovaras* G. [10], 2008	45 (20–90)^a^	47 (20–110)^a^	21	-	-
Ellakany M1. [22], 2013	67.3 ± 16.3^b^	68.6 ± 16.6^b^	-	3	8
Bessa SS [23], 2010	41.7 ± 14.7^b^	40.4 ± 15.6^b^	9	1	9
Tiwari S [12], 2013	36.11 ± 4.98^b^	34.22 ± 5.83^b^	8	3	5

Data are expressed as mean ± standard deviation or as numbers

^a^Median (range); ^b^mean ± standard deviation

**Table 5:** Meta-analysis of the secondary outcomes in 7 RCTs

Quantitative synthesis					Heterogeneity		Bias
Outcomes	OR (95 % CI)		z	*p*	I^2^	*P*	*P*
Operating Time	0.18	−0.06	1.47	*P* = 0.141	58 %	0.03	0.74
		0.43					
Post operative nausea and vomiting	0.43	0.26	3.35	*P* = 0.001	4 %	0.39	0.34
		0.7					
Post opertation Urinary retention	4.27	1.23	2.28	*P* = 0.022	0 %	1	0.96
		14.85					
Overall Morbidity	0.69	0.5	2.22	*P* = 0.027	0.00 %	0.71	0.91
		0.96

**Fig. 6:**
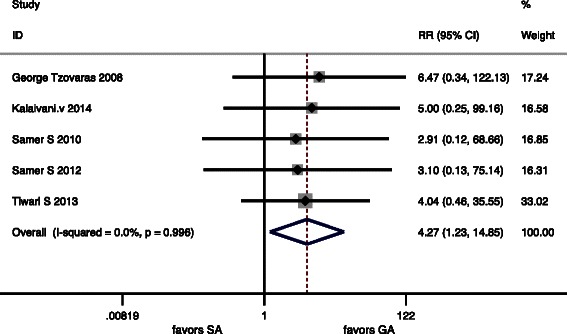
Forest plots for Urinary retention

**Fig. 7:**
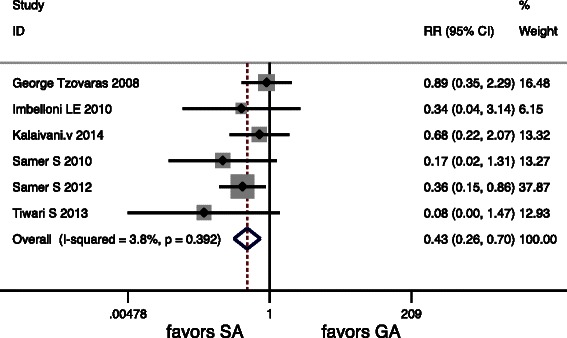
Forest plots for PONV

**Fig. 8:**
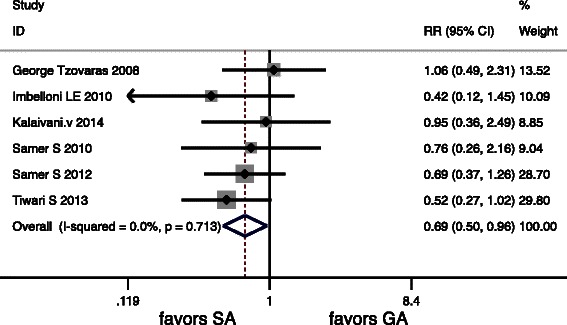
Forest plots for Overall Morbidity

Six articles included in the meta-analysis showed complication, but only one study did not give the exact figures of complication. There were also significant differences in PONV, Urinary retention and Overall Morbidity (*P* < 0.05) (Table 5). There were no deaths in any of the included RCTs. Forest graphs for postoperative complications are shown in (Figs. 6, 7 and 8).

### Test of heterogeneity

The results of heterogeneity testing are summarized in Tables 3 and 5. Significant heterogeneity existed in primary outcomes. We evaluated study quality and general characteristics of the included studies as potential sources of methodological and clinical heterogeneity. Meta-regressions were performed for postoperative pain scores to assess the potential reasons.

A standard four-trocar technique of LC was followed in all articles, and all selected studies met the following exclusion criteria: (a) patients with acute cholecystitis, and (b) previous upper abdominal surgeries. So subgroup analyses were conducted by stratifying study quality (high-quality *vs*. low-quality) to verify the accuracy of the meta-regression and assess the possible sources of heterogeneity. There were no significance sources of methodological and clinical heterogeneity identified by the meta-regression and subgroup analyses (Data not shown).

### Sensitivity analysis and publication bias

Sensitivity analysis was conducted to assess the effect of study quality. Only VAS from postoperative 6 to 8 h were affected by low quality of study (Data not shown). After low-quality studies were excluded, there was no statistically difference in VAS from postoperative 6 to 8 h. Statistical publication bias was detected for VAS in three time points, according to Egger’s test (*P* < 0.05) (Tables 3 and 5).

## Discussion

The less postoperative pain within 12 h and postoperative complications were found in SA group. There was no significant difference between SA group and GA group in regard to OT and postoperative pain in 24 h.

There were several limitations to this study. The meta-regression and subgroup analyses we performed did not account for all the sources of heterogeneity, which existed in the great majority of continuous variables (Tables 3 and 5). Random effects models were used when heterogeneity existed, although the stability of the pooled analyses could not be affirmed. There was also publication bias in some of the outcomes. One potential reason is that no withdrawals or dropouts were reported in the some of articles, and Jadad’s revised rating scale for the RCTs was low. Finally, we performed an electronic search and a manual search in order to identify any potentially relevant articles. We may have missed some meaningful articles, especially those not in English.

A major focus of this study was to determine which anesthesia method was associated with the least postoperative pain. In our meta-analyses, we found a significant difference in the VAS scores at postoperative 2 to 4 h and 6 to 8 h. The reduced pain in the SA group may be due to a persistent neuraxial blockade by SA. The postoperative VASs could be influenced by intraperitoneal pressure, use of local anesthetics, peritoneal irrigation, psychological factors and type of incision [26–28]. These factors could also contribute to heterogeneity. Although all of RCTs reported a postoperative pain score, different time points and methods were used (Table 4). The presence of heterogeneity and publication bias prevented identification of a superior anesthetic technique. Future prospective double-blind randomized controlled studies will need to address the issue of postoperative pain at different time points.

Although, recent studies have shown that laparoscopy in patients with regional anaesthesia may be tolerated well, shoulder tip pain can be a significant intraoperative problem. The reported incidence for intraoperative right-shoulder pain in previous studies requiring iv fentanyl administration ranged from 10 to 55.2 % [9–13]. Referred pain to right shoulder is probably due to irritation of diaphragm by the CO_2_ pneumoperitoneum [29]. In the our study, 92 patients (26.1 %) complained of shoulder pain.

### Intraoperative hypotension is another problem for LC

Under spinal anesthesia [3, 8] 74 (21 %) patients developed hypotension during operation in our study. Intravenous ephedrine injection solved this problem in all patients. In-traoperative hypotension is a common problem for patients undergoing LC under spinal anesthesia, but intravenous ephedrine injection is a very effective treatment.

In our study there was no statistically significant difference in mean operative time between the SA gand GA groups suggesting that SA in LC did not interfere either the adequacy of the surgical view or access thus not prolonging the operative time significantly.

Postoperative nausea and vomiting are relatively common after LC under general anesthesia [30]. In other series where LC was applied under spinal anesthesia, nausea and vomiting were not common [7, 8]. The surgical technique of LC was not different in spinal anesthesia compared to general anesthesia. Thus, the low incidence of nausea and vomiting seems to be related to the spinal approach.

In our meta-analyses, we found a significant higher incidence of Postoperative urinary retention in SA group. This is known to be related to regional anesthesia with rates of up to 20 % in some series [31].

We also found a significant difference in overall morbidity. Patients may have had less incidence of postoperative complications in SA group suggesting that LC under SA is safe.

## Conclusion

This study confirms the feasibility and safety of spinal anaesthesia as the sole anaesthesia technique for elective laparoscopic cholecystectomy. There was not enough data to support SA as the standard of care as it has small number of the cases. A large prospective double-blind randomized controlled trial comparing SA and GA in LC is needed to identify the best procedure.
